# Dental Legislation

**Published:** 1897-02

**Authors:** Geo. H. Wilson

**Affiliations:** Cleveland, Ohio


### THE DENTAL REGISTER.

VOL. LI.] FEBRUARY, 1897. [No. 2.

### COMMUNICATIONS.

### Dental Legislation.

BY GEO. H. WILSON, D. D. S., CLEVELAND, OHIO.

Read before the Ohio State Dental Society, December, 1896.

MR. PRESIDENT AND GENTLEMEN: In writing a paper on this subject, we make no claim to a scientific discourse, but shall endeavor to point out the deficiencies in our present law, and shall hope to evoke a discussion that will result in a more perfect one.

It is not necessary at this late day to make a plea for dental laws, as every commonwealth in this Union has dental legislation except the States of Idaho and Nevada, and the Indian Territory and Alaska. British America, Europe, Australia, Japan and some of the islands of the sea have more or less valuable dental enactments, also portions of South America and Africa.

A very natural inference is that dental legislation is a child of dental associations, but this is not necessarily so; Arizona and Wyoming, of our own country, have very good dental legislation, but no dental society (organized). Some foreign countries have more or less rigid examination laws, but no associations. The Argentine Republic is very rigid in requirements. Their students must have a preliminary education equivalent to our B. S. or A. M. before they can enter upon a two years' course of study (dental) in their University. A foreign educated dentist must pass the same examination as their own students before they will be permitted to practice, and this before a committee consisting of three "academicos" and five professors, the Dean of the Medical Department of the University presiding. Brazil has but one society, and no dental journal, but enacted its first dental law in 1854 and another in 1891. She requires all foreign-educated dentists to pass the examinations of her Examining Board. In 1894 Belgium, the Netherlands, Portugal, Servia, Spain and Turkey-in-Europe had laws regulating practice, but no dental societies.

From "The History of Dental and Oral Science in America," published in 1876, we quote the following paragraph:

"Curiously enough, the first State to pass a dental enactment in this country was Alabama, almost the poorest at the time, in skilled dentists, of any State in the Union. This legislation (probably the first ever had on the dental specialty), was somewhat anomalous, placing the keeping of dental interests entirely in the hands of the general surgeon and physician. The old and now well-known objections to such a course operated then much more actively than they do at present."

Dr. Chapin A. Harris, in commenting upon the Alabama law in the *American Journal of Dental Science*, says: "Much may be done, even in this way, but the true remedy lies in the general union of the educated dentists in a central association, aided and sustained by State societies. Such, acting with as much power from the State laws as surgeons and physicians have, will be able to make the profession honorable, respectable and useful."

While it is the duty of the State, and she does enact laws for the health, comfort and happiness of her citizens, it is also true that the controlling and restraining enactments may be of benefit to the individual members of a vocation, so that the reputable members of the calling may be united not only in fostering, but in improving the statutes. Hon. Charles G. Garrison, M. D., a member of the New Jersey bar, author of the article upon "Dental Jurisprudence," in the *American System of Dentistry*, says: "In the United States the spirit of the law favors the right of every man to practice any business or calling that he may elect, subject only to those State laws which are enacted for the protection of society from imposition and danger. Until within a few years this one principle of professional legislation has been all the law has contemplated. In all civilized countries there is special legislation for the children of unnatural parents, and those like Topsy. Pardon the comparison, but it conveys the idea.

From these few statements, and the known history of the profession, I believe every candid dentist will admit that the State is the primary mover in dental legislation, and that, by this legislative control, the best interests of society and the profession are subserved; also, that it is a mark of loyalty to the State and profession when the members of the profession exert themselves to enforce and improve the statutory measures.

The Alabama law, approved December 31, 1841, had four sections:

SECTION 1: Empowering the State Medical Boards to examine and license applicants to practice dentistry.

SEC. 2: Creating a penalty of $50 for each offense.

SEC. 3: Making all charges of a non-licensed dentist uncollectible.

SEC. 4: Requiring all practicing physicians, surgeons and dentists to have their licenses recorded in the office of the clerk of the county court.

New York was the next State to enact a dental law. A bill, composed of fifteen sections, was approved April 7, 1868. This bill was headed "An Act to Incorporate Dental Societies, for the Purpose of Improving and Regulating the Practice of Dentistry in this State." The bill provided for the formation of eight District Dental Societies, and a State Society. The State Society was empowered to appoint eight censors, one from each of the district societies, with power to examine, grant diplomas, and license the qualified applicants.

We quote Section 9 verbatim:

"SEC. 9. All dentists in regular practice at the time of the passage of this act, and all persons who shall have received a diploma from any dental college in this State, and all students who shall have studied and practiced dental surgery with some accredited dentist or dentists for the term of four years, shall be entitled to an examination by the board of censors. Deductions from such term of four years shall be made in each of the following cases: 1. If the student, after the age of sixteen, shall have pursued any of the studies usual in the colleges of this State, the period, not exceeding one year, during which he shall have pursued such studies, shall be deducted. 2. If the student, after the age of sixteen, shall have attended a complete course of lectures of any incorporated dental or medical college in this State, or elsewhere, one year shall be deducted."

This law was twice amended in '79, and again in '81, '89 and '92. Their present most excellent law was signed by their Governor May 12, 1895. This law repealed all of the amendments and all of the original law of '68 except that portion relating to the formation of the eight District and the State Societies.

Just thirty-one days after New York adopted her first dental law, Ohio approved her first dental enactment, May 8, 1868.

This bill had three salient sections:

1. Making it unlawful for any person to practice dentistry for a compensation without first having a diploma from a reputable dental college or a certificate of qualification from the State Dental Society or a local society auxiliary thereto.

2. Creating a penalty of $50 to $200.

4. Placing the prosecution in the Court of Common Pleas, and the fines to the credit of the common school funds.

On March 10, 1873, the bill was amended by striking out the words "or by any local society auxiliary thereto," and added these words, "that in all cases where any person has been continuously engaged in the practice of dentistry for a period of five years or more, such person shall be considered to have complied with the provisions of this act, and to the act to which this is amendatory."

This law remained upon the statute-books until April 8, 1892, when it was amended so as to create a Board of five Dental Examiners, appointed by the Governor, whose duty it shall be "to carry out the purposes and to enforce the provisions of this act." The law provides for the registering of all legal practitioners of dentistry; the issuing of certificates of registration to the graduates of any reputable dental college who may desire to practice dentistry within the State, to all persons in practice prior to July 4, 1889, and to any applicant for examination found proficient in the branches usually taught in a dental college. The law requires that the certificate of registration shall be kept in a conspicuous position in the possessor's place of business. It also makes it the duty of the prosecuting attorney of each county in the State to prosecute every case to final judgment whenever his attention shall be called to a violation of the law. The penalty for the violation of the provisions of the bill is $25 to $100 or be confined not less than ten days or more than one month in the county jail—or both.

This bill was again amended May 21, 1894; the amendments passed both branches of the Legislature the last day of their sessions. As the amendment is short, but pregnant, I will quote it in full:

"House Bill No. 651.—An Act to Provide for and Encourage a more Scientific Study of Dentistry:

"Section 1. *Be it enacted by the General Assembly of the State of Ohio*, That Section 4,404, of the Revised Statutes, as amended April 8, 1892 (87 O. I, p. 237) be supplemented as follows, with sectional numbering 4,404a, Sec. 4.404b; that for the purpose of this act, colleges of dentistry shall be considered reputable which are under State control or are organized, controlled and governed by a Board of Trustees, as provided by law for governing colleges of medicine, which possess buildings, by lease or otherwise, and equipments valued at not less than five thousand dollars, which have a graded course of study of not less than three years, the time of instruction in each year not less than six months, and which have a curriculum which includes anatomy, physiology, histology, pathology, chemistry, microscopy, materia medica, metallurgy; operative, mechanical and surgical dentistry.

"Sec. 2. This act shall take effect on and after its passage."

While it is impossible for the Governor to look after the interests of the various professions unaided, it is right that he should have the appointing power, because the primary interests are the State's, and he is the representative of the State; but the profession interested is the most capable of giving worthy advice. The second weak point in the law of '92 is, that while it makes it the duty of the prosecuting attorney to prosecute all offenders, it does not provide a means for procuring the evidence. The *despicable* portion of our law is the amendment of '94. It was conceived as a scheme, passed the Legislature by a scheme, and is a scheme from beginning to end to deceive. Let us analyze it: The claim is, "An act to provide for and encourage a more scientific study of dentistry." Section 4,404a has for its purpose defining what shall make a dental college reputable in this State, and not as the heading reads, "To provide for and encourage a more scientific study of dentistry." Conditions of reputability:

1. Under State control. This is a condition which does not exist in this State, but sounds well, so it is inserted.

2. Condition. "Organized, controlled and governed by a Board of Trustees, as provided by law, for governing colleges of medicine." This may, or may not, mean much. It is a very easy matter for a few designing men to operate together and secure the consent of a few more to permit the use of their names upon the Board of Trustees, provided they are not required to attend the meetings. Then it is quite easy to secure a few worthy people who will attend, but this class must be less in voting strength than the original interested parties; so this may, or may not, mean much. But this amendment has certain requirements: 1. That the institution shall possess, by lease or otherwise, buildings and equipments valued at not less than $5,000. We will suppose that the few men proposing to form a dental college have no money to buy, so they lease property valued at $5,000, at $100 or $500 a year, or $41.67 a month. The law requires that the school shall have a certain curriculum, but this must necessarily be on paper at first. It is not apparent that the veriest diploma-mill can be legalized in this State upon an expenditure of not more than $50 per month? What is this curriculum that is to so wonderfully uplift the dental profession, that the State of Ohio must have a special enactment? It is the list of subjects found in Section 4,404, Article 3, required of a non-college student, to be admitted to practice in the State. Why is this enacted into law as a distinctive mark of a reputable school? Now, what have we in this amendment for the advancement of dentistry not found in the law of '92? Nothing but the leasing of $5,000 worth of property, or associating with some institution that has leased that amount of property. I pronounce this amendment a deception and a fraud—an insult to the whole profession. Why should this be done?

The State has no need for educational institutions that require special enactments to make them reputable. The dental profession has no need for a school that is established for the purpose of giving occupation to a few men, whether fresh from college or older men that have not made a success otherwise, or as a money-making scheme for a few men, either medical or dental.

We now have nearly sixty so-called dental colleges in this country, and probably not less than 7,000 students attending them this year. Is it not apparent that we do not need more schools, but better ones? Is it not a fit subject for legislation? Should not all professional schools be under State control, and no new ones incorporated, unless it is apparent that the State is in need of greater facilities for giving professional training? Should the State not require that an organization, desiring a charter for a dental school, shall show that it will have suitable quarters and equipment equal to the best schools in the country and an endowment fund sufficient to secure competent teachers?

With the conditions, as described, before us, I believe that not only the State Society, but the whole profession is desirous of redeeming our State from being the inviting field for all kinds of dental quackery. We are practically helpless and something should be done at the earliest possible moment.

As a basis for discussion I suggest the following synopsis for a new dental bill:

1. A Board of four Dental Examiners, to consist of the State School Commissioner and three members of the dental profession, appointed by the Governor from nine nominees selected by the State Society. The duties of the Commissioner will be to inspect, at least once a year, the dental schools of the State; inspect the building and equipments, and examine the admission-credentials of each student and report his findings to the Governor. He is also to report all schools (dental) that have a standard equal to our own, and these schools only are to be recognized by this State. It shall be the duty of the dental members of the Board to examine, in the branches of study of a college curriculum, only such applicants as are graduates of the schools commended by the Commissioner and those persons who may desire to practice in the State, that have been legal practitioners in another commonwealth for not less than ten years. They shall license all successful candidates, keep a complete record of all licenses, prosecute all violators of the enactment and render an annual report to the Governor—the Board to be composed of men in no way connected with a dental school, except he may be an alumnus, and no two graduates from the same school.

2. *College Requirements.*—Every student for admission to a dental college of this State shall present credentials of not less grade than a common-school-teacher's certificate until the fall of 1900, when a high-school diploma or its equivalent shall be required. The college course shall consist of three scholastic years, of not less than seven months. At no time shall the standard of the State be less than that of the National Board of Dental Examiners. To secure a charter for a new dental college in the State the application shall have the approval of the Commissioner and two members of the Examining Board, and an affidavit of the possession of an endowment fund which shall be sufficient to render an annual income of not less than $2,000.

3. *Assessments, Fines and Compensations.*—Each applicant for examination shall pay an assessment of $20. Each violator shall be fined from $50 to $200, one-half of which shall go to the common-school fund of the county, and the other half to the treasury of the Dental Board. Each member of the Board shall be compensated in a sum not exceeding ten dollars a day and necessary mileage for time actually employed. An attorney may be employed by the Board to assist the prosecuting attorney, and be paid from the treasury of the Board.

Under these three headings all the items for a complete dental law can be grouped. This Society, recognizing the defeat of its bill before the Legislature last winter, should take action whether it desires to make farther effort in this direction or not.

If it is thought best, a committee should be appointed and empowered to proceed at once to draft a new bill, and also to organize the whole profession of the State.

I will close by giving a few facts gleaned from the various State laws.

THE APPOINTING POWER.

*New Jersey:* The State Society recommend five dentists to the Governor, whom he shall appoint.

*Alabama, Kentucky, and Louisiana:* The State Society.

*Colorado:* The Governor, by the consent of the Senate.

*District of Columbia:* Commissioners of the District.

*Indiana:* Governor, one; State Board of Health, one; State Society, three.

*New York:* Board of Regents, upon nomination of the State Society.

DENTAL COLLEGE DIPLOMA REQUIRED.

*Colorado:* Diploma or license from some other State.

*Minnesota:* Diploma and examination.

*Missouri, Nebraska, and Wyoming:* Require diploma for registration.

*New Jersey:* Diploma, or five years a registered pupil before examination.

*Washington:* Diploma, or ten years' practice before examination.

*New York:* Diploma, and examination by Board of Regents for license.

DEBARRED FROM SERVING ON BOARD.

*Massachusetts:* Any pecuniary association with a dental school.

*New York:* Any member of a faculty of a dental school.

Pennsylvanians will present a bill to their Legislature this winter, in which they desire to create a Dental Council consisting of the President of the State Society, the President of the

Board of Sanitation, and the Commissioner of Education. This Council is to act in conjunction with a Board of five Dental Examiners.

